# ITI consensus report on zygomatic implants: indications, evaluation of surgical techniques and long-term treatment outcomes

**DOI:** 10.1186/s40729-023-00489-9

**Published:** 2023-09-12

**Authors:** Bilal Al-Nawas, Tara Aghaloo, Carlos Aparicio, Edmond Bedrossian, Lawrence Brecht, Matthew Brennand-Roper, James Chow, Rubén Davó, Shengchi Fan, Ronald Jung, Peer W. Kämmerer, Vinay V. Kumar, Wei-Shao Lin, Chantal Malevez, Dean Morton, Justin Pijpe, Waldemar D. Polido, Gerry M. Raghoebar, Lambert J. Stumpel, Frank J. Tuminelli, Jean-Baptiste Verdino, Arjan Vissink, Yiqun Wu, Sepehr Zarrine

**Affiliations:** 1 Department of Oral and Maxillofacial Surgery, Plastic Operations, University Medical Center Mainz, Mainz, Germany; 2 Section of Oral and Maxillofacial Surgery, UCLA School of Dentistry, Los Angeles, CA USA; 3 Indiana University School of Dentistry, Indianapolis, USA; 4 Hepler Bone Clinic, ZAGA Center Barcelona, Barcelona, Spain; 5 Department of Oral and Maxillofacial Surgery University of the Pacific, Dugoni School of Dentistry, San Francisco, CA USA; 6 Department of Dental Medicine and Oral and Maxillofacial Surgery, Northwell Health, New Hyde Park, New York, USA; 7 Division of Prosthodontics and Restorative Dentistry, NYC College of Dentistry, New York, USA; 8 Department of Restorative Dentistry, Bristol Dental Hospital, Bristol, UK; 9 Brånemark Osseointegration Center, Hong Kong SAR, China; 10 Medimar International Hospital, Department of Implantology and Maxillofacial Surgery, Vithas Davó Instituto Dental, Alicante, Spain; 11 Second Dental Center, School of Medicine, Ninth People’s Hospital Affiliated to Shanghai Jiao Tong University, Shanghai, China; 12 Clinic of Reconstructive Dentistry, Center of Dental Medicine, University of Zurich, Zurich, Switzerland; 13 Oral Rehabilitation Center, Bangalore, India; 14 Department of Surgical Sciences, Odontology and Maxillofacial Surgery, Medical Faculty, Uppsala University, Uppsala, Sweden; 15 Department of Prosthodontics, Indiana University School of Dentistry, Indianapolis, USA; 16 Department of Oral and Maxillofacial Surgery, Clinique Saint-Jean, Brussels, Belgium; 17 Department of Oral and Maxillofacial Surgery, Special Care and Orthodontics, Erasmus Medical Center Rotterdam, Rotterdam, The Netherlands; 18 Department of Oral and Maxillofacial Surgery, Catharina Hospital, Eindhoven, The Netherlands; 19 Department of Oral and Maxillofacial Surgery, Indiana University School of Dentistry, Indianapolis, USA; 20 Department of Oral and Maxillofacial Surgery, University of Groningen, University Medical Center Groningen, Groningen, The Netherlands; 21 San Francisco, USA; 22 Department of Veterans Affairs, New York Harbor Healthcare System, New York, USA; 23 Hyères, France; 24 Department of Oral and Maxillofacial Surgery, University Medical Center Groningen and University of Groningen, Groningen, The Netherlands; 25 Saint-Dié-Des-Vosges, France

**Keywords:** Zygomatic implants, Atrophic maxilla, Edentulous, Survival, Complications

## Abstract

**Objectives:**

The aim of the ITI Consensus Workshop on zygomatic implants was to provide Consensus Statements and Clinical Recommendations for the use of zygomatic implants.

**Materials and methods:**

Three systematic reviews and one narrative review were written to address focused questions on (1) the indications for the use of zygomatic implants; (2) the survival rates and complications associated with surgery in zygomatic implant placement; (3) long-term survival rates of zygomatic implants and (4) the biomechanical principles involved when zygoma implants are placed under functional loads. Based on the reviews, three working groups then developed Consensus Statements and Clinical Recommendations. These were discussed in a plenary and finalized in Delphi rounds.

**Results:**

A total of 21 Consensus Statements were developed from the systematic reviews. Additionally, the group developed 17 Clinical Recommendations based on the Consensus Statements and the combined expertise of the participants.

**Conclusions:**

Zygomatic implants are mainly indicated in cases with maxillary bone atrophy or deficiency. Long-term mean zygomatic implant survival was 96.2% [95% CI 93.8; 97.7] over a mean follow-up of 75.4 months (6.3 years) with a follow-up range of 36–141.6 months (3–11.8 years). Immediate loading showed a statistically significant increase in survival over delayed loading. Sinusitis presented with a total prevalence of 14.2% [95% CI 8.8; 22.0] over a mean 65.4 months follow-up, representing the most common complication which may lead to zygomatic implant loss. The international experts suggested clinical recommendations regarding planning, surgery, restoration, outcomes, and the patient’s perspective.

## Introduction

Zygomatic implants (ZIs) were developed and introduced by Prof. P-I Brånemark in the late 1980s. They were originally designed to obtain stable prosthesis retention in patients with severe maxillary alveolar bone resorption or partial or complete loss of the maxillary bone secondary to oncologic resection, who were not suitable for conventional dental implant placement. The original Brånemark protocol included one implant in each zygoma, traversing the sinus, combined with two to four anterior conventional implants. Since then, many modifications to zygomatic implant designs, surgical approaches and loading protocols have been documented in the literature. For cases without adequate anterior maxillary bone, the quad zygomatic implant concept was introduced, where two zygomatic implants are inserted on each side, providing acceptable antero-posterior implant positioning for force distribution. Over the past 20 years, indications for zygomatic implants have evolved to include severe posterior maxillary resorption with insufficient bone for conventional implant placement, with or without previously failed implant or bone graft treatment. Other indications described in the literature include maxillary deficiency secondary to cleft palate, failed conventional implant therapy, unsuccessful bone grafting or refusal to undergo bone grafting. Patients that underwent complete or partial maxillectomy secondary to benign or malignant tumor resections are still one of the main reported uses for zygomatic implants, assisting in supporting obturators and/or removable prostheses.

Although still a complex procedure with significant surgical risk and potential for complications, the use of zygomatic implants has grown exponentially, with documented high survival rates.

What is unclear in the literature is when zygomatic implants should be utilized instead of traditional bone grafting procedures or other graftless alternatives. Many papers cite “severe maxillary atrophy” or “atrophic maxilla” without defining the degree of bone resorption or available bone. Moreover, there have been many advances with conventional implants, where improved implant surfaces, materials, and strong evidence behind reduced diameter and short implants may allow for their expanded use in atrophic situations.

However, the possibility of shortened treatment time, including immediate loading, engagement of stable cortical bone in the zygoma, and lack of a need for grafting has influenced the decision to utilize zygomatic implants to rehabilitate atrophic edentulous maxillae with an implant-supported prosthesis.

The aim of this Consensus Workshop was to provide Consensus Statements and Clinical Recommendations for the use of zygomatic implants as well as to identify topics for future research.

## Methodology

On March 24, 2023, the ITI held a consensus workshop on zygomatic implants in order to look at and evaluate the current literature towards reaching a consensus and providing evidence-based recommendations for the safe use of zygomatic implants. The meeting, held in Frankfurt, Germany, was chaired by Bilal Al-Nawas.

Under the lead of the Consensus Workshop Steering Committee (Table 1), 25 international experts in the area of zygomatic implants were identified and invited to participate. In an online kick-off meeting 3 working groups were formed, each of which defined a PICO question for a systematic review and nominated the main author for its review paper. All participants disclosed their potential conflicts of interest to the Steering Committee.

**Table 1:** The 4 review papers that formed the basis for discussion during the ITI Consensus Workshop and the working groups

Steering Committee: Tara Aghaloo, Bilal Al-Nawas and Ronald Jung			
Group 1: Indications Group Leader: Tara Aghaloo			
	Title of review	Authors	Working Group on site
Paper 1 [1]	Indications for zygomatic implants: a systematic review	Waldemar D. Polido, Agustin Machado-Fernandez, Wei-Shao Lin, Tara Aghaloo	Tara Aghaloo, James Chow, Wei-Shao Lin, Justin Pijpe, Waldemar D. Polido, Lambert Stumpel, Frank Tuminelli, Jean-Baptiste Verdino
Group 2: Surgical techniques Group Leader: Bilal Al-Nawas			
	Title of review	Authors	Working Group on site
Paper 2 [2]	Evaluation of surgical techniques in survival rate and complications of zygomatic implants for the rehabilitation of the atrophic edentulous maxilla: a systematic review	Peer W. Kämmerer, Shengchi Fan, Carlos Aparicio, Edmond Bedrossian, Rubén Davó, Dean Morton, Gerry M. Raghoebar, Sepehr Zarrine, Bilal Al-Nawas	Bilal Al-Nawas, Carlos Aparicio, Edmond Bedrossian, Rubén Davó, Shengchi Fan, Peer W. Kämmerer, Dean Morton, Gerry M. Raghoebar, Sepehr Zarrine
Paper 3 [3]	Zygoma implant under function: biomechanical principles clarified	Edmond Bedrossian, John Brunski, Bilal Al-Nawas, Peer W. Kämmerer	
Group 3: Long-term treatment outcomes Group Leader: Ronald Jung			
	Title of review	Authors	Working Group on site
Paper 4 [4]	Long-term treatment outcomes with zygomatic implants: a systematic review and meta-analysis	Matthew Brennand Roper, Arjan Vissink, Tom Dudding, Alex Pollard, Barzi Gareb, Chantal Malevez, Thomas Balshi, Lawrence Brecht, Vinay Kumar, Yiqun Wu, Ronald Jung	Ronald Jung, Lawrence Brecht, Matthew Brennand-Roper, Vinay Kumar, Chantal Malevez, Arjan Vissink, Yiqun Wu

In preparation for the consensus workshop, the main authors prepared three systematic review papers and a narrative review paper together with their co-authors, which were submitted to the *International Journal of Implant Dentistry* before the workshop and went through a peer review process. During the consensus workshop, based on the four review papers, the three working groups (Table 1) prepared Consensus Statements, Clinical Recommendations and recommendations for future research which are published in this consensus report.

The International Team for Implantology (ITI) is a non-profit association of professionals in implant dentistry. The mission of the ITI is “to serve the dental profession by providing a growing global network for life-long learning in implant dentistry through comprehensive quality education and innovative research for the benefit of the patient”.

### Preparing the reviews

Each of the three working groups was given a topic by the Steering Committee, and the main author asked to produce a systematic review paper on that topic. The main author first performed a literature search based on a PICO question that was formulated by his working group and then summarized the findings reported in the literature in a systematic review paper. The paper contains only the information that could be extracted from the literature and its discussion. When interpreting the results, consideration should be given to limitations around the quality of reporting. Although the inclusion criteria of primary studies were clear, it was often unclear as to whether there had been consecutive and/or complete inclusion of the participants. As such, inclusion bias may have played a role in the selection of participants selected for studies. Heterogeneity in reporting success was notable across the studies, which challenged comparisons.

The participants of each working group had the opportunity to comment on their group’s systematic review paper in writing according to a structured written Delphi process. As it became obvious that for the surgical procedures not all aspects for the consensus process can be gathered in a systematic review, an additional narrative review was added. These articles are intellectual property of the respective authors and do not necessarily reflect a group consensus.

### Workshop

At the 1-day consensus workshop, each working group formulated the Consensus Statements, Clinical Recommendations and recommendations for future Research based on the findings of the group’s systematic review as the starting point for the deliberations and discussions. Where insufficient scientific evidence was available in the literature to formulate a Consensus Statement and/or a Clinical Recommendation, an “expert consensus” was reached based on the clinical experience (opinions) of the group members. All considerations stemming from the group discussions were then presented to and discussed by the entire plenary formed of all workshop participants. Once consensus was reached, the recommendations were merged into this overarching consensus report.

The Consensus Statements, Clinical Recommendations and recommendations for future research were developed from the reviews listed below that assessed (1) the indications for the use of zygomatic implants; (2) the survival rates and complications associated with surgery in zygomatic implant placement; (3) long-term survival rates of zygomatic implants; and (4) the biomechanical principles involved when zygoma implants are placed under functional loads.

**Systematic review paper:** Indications for zygomatic implants: a systematic review [1].

The purpose of this systematic review was to assess the evidence regarding the indications for placement of zygomatic implants to rehabilitate edentulous maxillae.

**Systematic review paper:** Evaluation of surgical techniques in survival rate and complications of zygomatic implants for the rehabilitation of the atrophic edentulous maxilla: a systematic review [2].

The purpose of this systematic review was to assess the outcome [zygomatic implant (ZI) survival] and complications of the original surgical technique (OST) and an Anatomy-Guided approach (AGA) in the placement of ZI in patients with severely atrophic maxillae.

**Systematic review paper:** Long-term treatment outcomes with zygomatic implants: a systematic review and meta-analysis [4].

The purpose of this study was to perform a systematic review with meta-analysis on the long-term survival rates of zygomatic implants (ZI). ZI success, prostheses survival and success, sinus pathology and patient-reported outcomes were also investigated.

**Narrative review paper:** Zygoma implant under function: biomechanical principles clarified [3].

The purpose of this review was to illustrate the biomechanical principles involved when zygoma implants are placed under functional loads.

For ease of reference the Consensus Statements, Clinical Recommendations and recommendations for future research were grouped by topic.

## Consensus statements

### Indications

#### Consensus statement 1

Zygomatic implants are indicated in cases with maxillary bone atrophy or deficiency (118 patients), unsuccessful previous treatments with grafts and/or implants (34 patients), avoidance of staged bone graft procedures (29 patients) and conditions that may complicate traditional bone grafting procedures, such as benign cysts and trauma (5 patients).

This statement is supported by 10 publications reporting on 209 patients and 622 implants.

It is based on the systematic review by Polido et al. [1].

#### Consensus statement 2

Zygomatic implants are an alternative when the maxillary bone is completely or partially absent, secondary to resection, trauma, or congenital defects.

This statement has a moderate level of evidence, it is supported by 3 papers (23 patients) reporting on the use of zygomatic implants in these situations.

It is based on the systematic review by Polido et al. [1].

#### Consensus statement 3

Zygomatic implants are an alternative when the maxillary bone is completely or partially absent, secondary to failure of previously placed implants and/or bone grafts.

This statement is supported by six articles reporting on 34 patients utilized as a rescue alternative in cases of failure of bone grafts and previous implants, and expert opinion.

It is based on the systematic review by Polido et al. [1].

### Survival

#### Consensus statement 4

This statement is based on 24 studies reporting treating 918 patients survival rates of ZI ranged between 90.3% and 100% after a follow-up of 6 months. There are many factors potentially altering the final outcomes.

It is based on the systematic review by Kämmerer et al. [2].

#### Consensus statement 5

Zygomatic implants are an evidence-based alternative to support fixed or removable prostheses to restore partially or completely edentulous maxillae, allowing high survival rates when splinted to other implants.

This statement is based on data collected from the papers included in this review, that showed a mean survival rate of 97% (89–100%) in a mean follow-up period of 28.5 months (range 12–162 months).

It is based on the systematic review by Polido et al. [1].

#### Consensus statement 6

The mean zygomatic implant survival was 96.2% [95% CI 93.8; 97.7] over a mean follow-up of 75.4 months (6.3 years) with a follow-up range of 36–141.6 months (3–11.8 years).

This statement is based on a meta-analysis of 18 case series reports, which included a total of 1349 ZIs placed in 623 patients.

It is based on the systematic review by Brennand Roper et al. [4].

### Quad zygoma

#### Consensus statement 7

The quad zygomatic implant approach (two zygomatic implants bilaterally placed) can be indicated as an alternative when conventional implants cannot be placed in the posterior and anterior maxillary regions, and grafting alternatives are not feasible, predictable, or preferred by patients. In this situation all implants should be splinted.

This statement is supported by 7 publications reporting on 107 patients with quad zygoma approach.

It is based on the systematic review by Polido et al. [1].

### Loading protocol

#### Consensus statement 8

ZI survival for immediate Loading protocols were 98.1% [95% CI 96.2; 99.0] over a mean of 73.6 months follow-up.

This statement is based on a meta-analysis of 7 case series reports, which included 458 ZIs.

It is based on the systematic review by Brennand Roper et al. [4].

#### Consensus statement 9

Mean survival prevalence for delayed load protocols was 95% [95% CI 91.7; 97.1] over a mean of 69.3 months follow-up.

This statement is based on a meta-analysis of 7 case series reports, which included 535 ZIs.

It is based on the systematic review by Brennand Roper et al. [4].

### Surgical risks

#### Consensus statement 10

Fifteen citations report transient infraorbital nerve (V2) paresthesia, 15 studies report oro-antral communications, and 16 report peri-abutment soft tissue recession and/or hyperplasia. In six cases, a fractured ZI was described. Failure is mostly seen within the first 6 months.

This statement is based on the systematic review by Kämmerer et al. [2].

#### Consensus statement 11

Intraoperative-specific complications of ZI malposition, orbital cavity penetration (reported in four cases in three studies; in one case with lateral rectus muscle damage), and subcutaneous peri-malar emphysema are reported.

This statement is based on the systematic review by Kämmerer et al. [2].

### Failures

#### Consensus statement 12

A higher failure incidence may occur within the first year (2%) compared to that of subsequent years (0.5%/year).

This statement is based on a meta-analysis of 17 case series reports, which included a total of 1247 ZIs.

It is based on the systematic review from by Brennand Roper et al. [4].

#### Consensus statement 13

The overall annual failure incidence of ZIs was 0.7%, with follow-up times ranging from 36 to 141.6 months.

This statement is based on a meta-analysis of 18 case series reports, which included a total of 1349 ZIs placed in 623 patients.

It is based on the systematic review by Brennand Roper et al. [4].

#### Consensus statement 14

Reasons for failure within the first year were related to a failure to integrate or subsequent loss of integration/stability. Failure within the subsequent years was related to loss caused by biological or mechanical complications.

This statement is based on a meta-analysis of 18 case series reports, which included a total of 1349 ZIs placed in 623 patients.

It is based on the systematic review by Brennand Roper et al. [4].

### Sinusitis

#### Consensus statement 15

Sinusitis is the most commonly reported biological complication related to ZI therapy. Sinusitis is reported to be the most common complication which may lead to ZI implant loss. There is no clear relationship between sinusitis and ZI survival.

Sinusitis presented with a total prevalence of 14.2% [95% CI 8.8; 22.0] over a mean of 65.4 months follow-up. The prevalence of sinusitis ranged from 2.8% [95% CI 0.1; 14.5] to 36.4% [95% CI 20.4; 54.9] from 36 to 141.6 months of mean follow-up.

Disease was diagnosed clinically, radiographically, using patient-reported questionnaires, or via combined methods.

This statement is based on descriptive data from 11 case series reports in 409 patients.

It is based on the systematic review by Brennand Roper et al. [4].

#### Consensus statement 16

Maxillary sinusitis was the most reported complication. Pooled incidence rates for sinusitis in the original surgical technique (OST) were 9.5% (0–37.5%), and in Anatomy-Guided were 4.4% (0–11.8%). In most cases, sinusitis was reported by the literature without differentiation between symptomatic and asymptomatic cases.

This statement is based on descriptive data of 864 patients.

It is based on the systematic review by Kämmerer et al. [2].

#### Consensus statement 17

Sinusitis may be successfully treated. When sinusitis was diagnosed, successful treatment with antibiotics and/or via a surgical meatotomy was reported with no further consequences.

This statement is based on descriptive data from 7 case series reports in 22 patients.

It is based on the systematic review from Brennand Roper et al. [4].

### Biological complications

#### Consensus statement 18

10 of 18 studies reported factors related to the biological complications of ZI therapy.

Among them, one study, investigating two groups of 10 patients (20 in total), reported a prevalence of peri-implant ZI mucositis at 13.1% (3.7–41%) within an atrophic group and 39.7% (9.7–91.7%) in an oncologic group over a group mean follow-up of 39.9 months (± 19.5).

One study reported recession exposing 2–3 threads in 14% of ZI implants (*n* = 6 of 43) in 25 patients over 72 months of mean follow-up (48–72).

One study reported infective soft tissue dehiscences affecting 6 of 67 ZI (9%) in 33 patients with a mean follow-up of 141.6 months (range 109–198).

This statement is based on the systematic review by Brennand Roper et al. [4].

### Technical complications

#### Consensus statement 19

Technical complications for ZI supported reconstructions include fracture of the metal substructure, chipping or loss of the veneering material (ceramic or acrylic), and abutment or screw fracture and/or loosening.

3 metal framework fractures occurred in 43 prostheses. 77 episodes of veneering acrylic loss occurred in 228 prostheses. 28 episodes of veneering ceramic loss occurred in 141 prostheses. 8 episodes of prosthetic tooth loss occurred in 116 prostheses. 29 episodes of screw or abutment loosening occurred in 323 prostheses. 15 episodes of screw or abutment fracture occurred in 136 prostheses.

This statement is based on descriptive data from 10 case series reports including 400 prostheses with a mean follow-up ranging from 36 to 120 months.

It is based on the systematic review by Brennand Roper et al. [4].

#### Consensus statement 20

The mean prosthesis survival supported by ZIs was 94% [95% CI 88.6; 96.9] at 76.0 months of mean follow-up. Prostheses constituted fixed and removable designs, with materials including resin and ceramic superstructures on metal substructures.

This statement is based on a meta-analysis of 9 case series reports, which included 304 ZI supported prostheses.

It is based on the systematic review by Brennand Roper et al. [4].

### Patient-reported outcome measures

#### Consensus statement 21

Patients reported an increase in satisfaction using a range of patient-reported outcome measures (PROMs) when rehabilitated with ZI supported reconstructions.

This statement is based on descriptive data from 7 case series reports in 266 patients using OHIP14, OHIP EDENT, Likert, and subjective questioning assessment tools.

It is based on systematic review by Brennand Roper et al. [4].

## Clinical recommendations

### Planning

#### Clinical recommendation 1

##### Who should perform zygomatic implant treatment?

Zygomatic implants are considered a complex treatment. The success of the treatment is highly dependent on the clinician skill and experience. There is a need for surgical and restorative expertise to address all potential difficulties and complications.

This clinical recommendation is based on the systematic review from Polido et al.

It is based on the systematic review by Polido et al. [1].

#### Clinical recommendation 2

##### Who is a candidate for zygomatic implants?

Zygomatic implants are an evidence-based alternative to support fixed or removable prostheses to restore partially or completely edentulous maxillae, allowing high survival rates when splinted to other implants.

Zygomatic implants are an alternative when the maxillary bone is completely or partially absent, secondary to resection, trauma or congenital defects.

Zygomatic implants are an alternative when the maxillary bone is completely or partially absent, secondary to failure of previously placed implants and/or bone grafts.

This clinical recommendation is based on the systematic review by Polido et al. [1].

#### Clinical recommendation 3

##### What diagnostic tools are recommended to assess the surgical field?

A CT/CBCT including the midface, allowing for 3D assessment of the maxillary and zygomatic bone volume and sinus health should be obtained.

Preoperative evaluation for a lack of existing sinus pathologies is recommended.

The use of specific software for planning, including the image of the planned prostheses and 3D anatomic models is an option.

This clinical recommendation is based on the systematic review by Polido et al. [1].

#### Clinical recommendation 4

##### What is the degree of maxillary atrophy to consider zygomatic implants?

Objective criteria should be utilized to determine the amount of bone atrophy. A 3D assessment of the maxillary and zygomatic bone volume is recommended.

The most cited anatomical classification is Cawood and Howell (1988), with class IV, V and VI.

Each site should be individually analyzed, and treatment options should be discussed with the patients, considering the risks, benefits, the final prosthetic outcome, total treatment time, long-term outcomes and patients preference and conditions.

This clinical recommendation is based on the systematic review by Polido et al. [1].

#### Clinical recommendation 5

##### Can I consider zygomatic implants for maxillofacial defects?

Zygomatic implants in maxillofacial rehabilitation cases have additional complexity and considerations. Factors such as surveillance of malignant disease, radiation, bone and soft tissues quality and quantity, patient compliance should be considered.

This clinical recommendation is based on the systematic review by Polido et al. [1].

### Surgery

#### Clinical recommendation 6

##### Can I place zygomatic implants at the same time as dental extractions?

Factors such as presence of infection, hard and soft tissue quality and quantity, clinician experience and patient preference should be considered.

Risks may be increased when performing zygomatic implants at the same time as tooth extractions.

This clinical recommendation is based on the systematic review by Polido et al. [1].

##### What is the role of guided surgery or dynamic navigation for insertion of zygomatic implants?

Direct visualization of the surgical field is paramount to avoid disorientation and anatomical complications (f.e. to the orbital cavity or the infra-temporal fossa).

This clinical recommendation is based on expert opinion.

#### Clinical recommendation 7

##### Should the sinus membrane be elevated (“preserved”) for insertion of zygomatic implants?

Neither the literature nor expert consensus on preserving the sinus membrane for ZI placement exists.

This clinical recommendation is based on expert opinion.

### Restoration

#### Clinical recommendation 8

##### Do specific loading protocols have an influence on the long-term outcomes of zygomatic implant therapy?

ZI survival rates appear to be slightly higher for immediate over delayed loading protocols subject to adequate primary implant stability. Immediate loading also confers benefits to the patient through immediate functional rehabilitation. However, delayed loading techniques are also clinically acceptable.

This clinical recommendation is based on the systematic review by Brennand Roper et al.

#### Clinical recommendation 9

##### When zygomatic implants are used, what type of prosthesis can be utilized?

Once generally accepted restorative concepts for implant-supported-prosthesis are followed, removable or fixed restorations can be considered, provided that all implants are splinted.

Factors to be considered include prosthetic material, esthetic factors (e.g., lip support, smile line), condition of the opposing dentition, available space for the prosthesis, planned implant distribution, presence and length of cantilever, space available for hygiene and maintenance, proper abutment selection and timing of implant platform position, patient preference and compliance.

This clinical recommendation is based on the systematic review by Polido et al. [1].

### Outcomes

#### Clinical recommendation 10

##### What are the long-term therapeutic advantages of zygomatic implants?

Current survival data support the use of zygomatic implants as a long-term therapeutic option.

ZIs present an opportunity to rehabilitate patients who lack either the desire to undergo extensive augmentation procedures, or lack the anatomical structures required to deliver conventional implant therapy in the maxilla.

ZIs may confer treatment time benefits to patients due to the possibility of immediacy in reconstruction.

This clinical recommendation is based on the systematic review by Brennand Roper et al.

#### Clinical recommendation 11

##### How does zygomatic implant survival perform long-term when compared to conventional implants?

Survival of ZIs appear to be comparable to conventional implants when used for reconstruction of the atrophic maxilla. This includes techniques such as short implants, tilted implants, and implants placed in grafted sinuses. With this in mind, ZIs may be considered as an option to support maxillary reconstructions.

This clinical recommendation is based on the systematic review by Brennand Roper et al.

#### Clinical recommendation 12

##### What is the long-term performance of zygomatic implant-supported reconstructions?

Within the context of long-term data survival analyses, ZI reconstructions are comparable to, and have similar survival characteristics to reconstructions supported by conventional implants.

They are subject to similar mechanical complications.

Although no additional technical considerations are required, ZI reconstruction should be considered as a complex procedure.

This clinical recommendation is based on the systematic review by Brennand Roper et al.

#### Clinical recommendation 13

##### What are the long-term mechanical complications associated with zygomatic implants?

The most common mechanical complications include ZI prosthesis abutment or screw fracture, abutment or screw loosening, and chipping or loss of the veneering acrylic or ceramic materials. These complications may occur whether the ZI reconstructions are splinted to conventional implants or supported by ZIs alone, through a Quad Zygomatic implant approach.

ZI fracture or reconstruction framework fracture have been reported as rare complications.

In light of these findings, conventional prosthetic techniques to mitigate such factors are recommended.

This clinical recommendation is based on the systematic review by Brennand Roper et al.

#### Clinical recommendation 14

##### What are the long-term biological risks associated with zygomatic implants?

The most reported long-term biological complication was sinusitis. This may be successfully treated through antibiotic and/or surgical interventions. If these therapies are unsuccessful, the ZI may be lost. Oro-antral communications, peri-implant infection of the soft tissues, peri-implant mucositis, bleeding on probing and increased probing pocket depths have also been reported. Patient education in oral hygiene maintenance is paramount.

This clinical recommendation is based on the systematic review by Brennand Roper et al.

#### Clinical recommendation 15

##### How should sinus infections in relation to zygomatic implants be treated?

Sinus infections are generally treated with antibiotics with a satisfactory resolution. In the absence of resolution, refractory maxillary sinus infections may need exploration of the patency of the osteo-meatal complex and other paranasal sinuses.

This clinical recommendation is based on expert opinion.

### Patient’s perspective

#### Clinical recommendation 16

##### Do patients perceive a long-term benefit from the zygomatic implant treatment experience?

Most patients report an increase in oral health-related quality of life and satisfaction with the treatment outcome.

This clinical recommendation is based on the systematic review by Brennand Roper et al.

#### Clinical recommendation 17

##### Are unique challenges faced by patients receiving zygomatic implants and their reconstructions?

Zygomatic implants and their reconstructions may require a higher level of professional maintenance. There are also limitations on the range of acceptable masticatory loads. Patients’ expectations need to be managed in line with the biological and technical complexities faced by zygomatic implant therapies.

This clinical recommendation is based on the systematic review by Brennand Roper et al.

## Recommendations for future research

### Recommendation 1 for future research

Standardized data sets for reporting outcomes for ZI research should be developed to allow systematic data analysis.

### Recommendation 2 for future research

Evaluate the influence of zygomatic implants’ geometry, surface properties, emergence position, abutment profile on complication rates.

### Recommendation 3 for future research

RCTs comparing the management of sinusitis or oro-antral communications when utilizing ZI therapy.

### Recommendation 4 for future research

Develop objective criteria to determine when zygomatic implants can be indicated. This can be achieved by:

- Develop a new classification of maxillary atrophy based on a three-dimensional analysis of edentulous maxillae; retrospective, multi-center analysis of CBCTs.
- Develop a peer-reviewed risk assessment analysis, in a decision tree format, to assist in determining if the use of zygomatic implants could be advantageous over other alternatives.

### Recommendation 5 for future research

Randomized Clinical trials comparing zygomatic implants vs short implants vs staged grafts vs. distally tilted implants, for rehabilitation of the completely edentulous maxilla.

### Recommendation 6 for future research

RCTs comparing restorative materials used for fixed and removable reconstructions supported by ZIs.

### Recommendation 7 for future research

Study the effect of opposing dentition and occlusal forces on zygomatic implant survival rates.

### Recommendation 8 for future research

Long-term and larger multi-center case series on the use of zygomatic implants to rehabilitate partial or complete loss of the maxilla, secondary to congenital, trauma and resection-related defects.

### Recommendation 9 for future research

Studies on indications and outcomes for unilateral fixed prostheses supported by at least one zygomatic implant, splinted to conventional implants.

## Data Availability

Protocols of the consensus meeting are available.

## Abbreviations

3D: Three-dimensional
CBCT: Cone beam computed tomography
CT: Computed tomography
OST: Original surgical technique
PICO: Patient/Population, Intervention, Comparison, Outcomes
PROMs: Patient-Reported Outcome Measures
RCT: Randomized controlled trial
OHIP: Oral Health Impact Profile
OHIP EDENT: Oral Health Impact Profile for Edentulous
ZI: Zygomatic implant

## References

[1] Polido WD, Machado-Fernandez A, Lin WS, Aghaloo T (2023). Indications for zygomatic implants: a systematic review. Int J implant Dent.

[2] Kammerer PW, Fan S, Aparicio C, Bedrossian E, Davo R, Morton D (2023). Evaluation of surgical techniques in survival rate and complications of zygomatic implants for the rehabilitation of the atrophic edentulous maxilla: a systematic review. Int J Implant Dent.

[3] Bedrossian E, Brunski J, Al-Nawas B, Kammerer PW (2023). Zygoma implant under function: biomechanical principles clarified. Int J Implant Dent.

[4] Brennand Roper M, Vissink A, Dudding T, Pollard A, Gareb B, Malevez C (2023). Long-term treatment outcomes with zygomatic implants: a systematic review and meta-analysis. Int J Implant Dent.

